# Delabelling multiple antibiotic allergy: Practical issues

**DOI:** 10.3389/falgy.2023.1156137

**Published:** 2023-03-16

**Authors:** Philip Hei Li, Bernard Yu-Hor Thong

**Affiliations:** ^1^ Division of Rheumatology and Clinical Immunology, Department of Medicine, Queen Mary Hospital, The University of Hong Kong, Hong Kong, China; ^2^ Department of Rheumatology, Allergy and Immunology, Tan Tock Seng Hospital, Singapore, Singapore

**Keywords:** penicillins, allergy, drug, antimicrobial stewardship, antibiotic

## Abstract

With the growing incidence of multi-drug resistant organisms, delabelling incorrect antibiotic allergies has become an integral part of antimicrobial stewardship worldwide. For example, around 90% of penicillin allergy labels are found to be inaccurate following a full allergy work-up, which deprive patients the use of effective first-line penicillin antibiotics and increase the risk of antimicrobial resistance with the use of other extended spectrum non-penicillin antimicrobials. Significant numbers of adult and paediatric patients over time are labelled with multiple penicillin and non-penicillin antibiotic allergies often during inappropriate antimicrobial use, resulting in a label of “multiple antibiotic allergy”. In contrast to delabelling penicillin allergy where oral direct provocation tests can be used for low-risk, mild reactions, and sensitivity/specificity/positive and negative predictive values of skin tests have been demonstrated, diagnostic tests for multiple antibiotic allergy often require the use of a combination of in-vivo and in-vitro tests across different antimicrobial classes for evaluation. Shared decision making with patients and informed consent are also needed when prioritising which drugs to delabel first, balancing the risks, benefits of testing vs. interim use of alternative antibiotics. Similar to delabelling penicillin allergy, the cost-effectiveness of delabelling multiple drug allergies is unknown.

## Introduction

With the growing incidence of multi-drug resistant organisms, delabelling incorrect antibiotic allergies has become an integral part of antimicrobial stewardship worldwide. Often patients have allergic and non-allergic adverse drug reactions inappropriately labelled as “drug allergy”, many incorrectly labelled, with little appreciation of future implications. For example, patients may be incorrectly labelled with multiple antibiotic allergies which severely limits future antibiotic prescription, especially should patients require antibiotics to treat acute infections, pre-operative or pre-procedural antimicrobial prophylaxis or long-term antimicrobial suppressive therapy (1). The terms multiple drug hypersensitivity syndrome [MDHS and multiple drug intolerance syndrome (MDIS) are distinct entities which have been clearly defined in the literature] (2–5). The benefits of antibiotic allergy delabelling is best exemplified with beta-lactam (in particular penicillin) allergy, which is often incorrectly and over-diagnosed (6–9). There has been much interest in programs to delabel suspected penicillin allergy both in adults and children, in particular in antimicrobial stewardship programs to prevent widespread use of alternative broad spectrum non-penicillin antibiotics which may lead to antimicrobial resistance and use of agents that may not be as effective as penicillins (10–13). Clinical pharmacist and nurse-led allergist supervised delabelling programs for low-risk index reactors have been shown to be safe, effective and potentially scalable (13, 14). Guidelines for penicillin allergy delabelling services in children and adults by non-allergists working in hospital settings, networked with a specialist allergy immunology service for advice and support, have also been published (15–17).

However, unlike with delabelling single penicillin allergies, the approach to patients labelled with multiple suspected drug allergies can be a complex issue. The cost-effectiveness of delabelling allergies to multiple antibiotic classes is also less clear-cut in contrast to delabelling a specific drug like penicillin (18, 19). In this review, we discuss the practical issues and challenges associated with delabelling multiple antibiotic allergy in contrast to what has been learnt from well-established penicillin allergy delabelling programs.

## Nomenclature and definitions

Multiple drug allergy refers specifically to individuals who have been diagnosed with probable or definite immune-mediated drug hypersensitivity reactions (DHR) based on a combination of corroborative/ consistent history, physical signs, in-vitro and/or in-vivo tests. This entity may be synonymous with MDHS although MDHS includes both immune- and non-immune mediated hypersensitivity reactions. In contrast, MDIS refers to intolerance to 3 or more chemically unrelated drugs (1). MDIS is not immune-mediated and has no defined mechanism responsible for the adverse reactions or claimed intolerance to medications. Thus the term multiple antibiotic allergy refers to patients with immune-mediated hypersensitivity to 2 or more antibiotic drug classes.

### Risk stratifying the index drug allergy episode

The history of any danger or “high risk” features in the index reaction is important in risk stratifying low-risk vs. non-low risk penicillin allergy patients (20). In practice, it is often challenging to differentiate patients with MDHS and MDIS to multiple antibiotic classes as the history is often remote (especially in the elderly or where vague drug allergy labels originated in childhood or early adulthood) and difficult to verify. Nonetheless, it is unlikely that a severe reaction (e.g., Stevens Johnson syndrome/toxic epidermal necrolysis, drug hypersensitivity syndrome or anaphylaxis) in the index history is missed unless there the patient has no recollection of hospitalization for the serious drug reaction, especially in elderly patients with cognitive impairment, or where no immediate family members witnessed or are able to recall the event either. Machine learning using datasets derived from electronic medical records and other digital assessment tools may in future help facilitate classification of index adverse drug reactions and risk assessment (21, 22). Structured and validated clinical decision tools or guidance, such as PEN-FAST, are straightforward and have also been demonstrated to aid with risk stratification (23).

## Drug reactions with similar stereotypical reactions

In individuals with multiple antibiotic allergy, one needs to consider if these patients truly have MDHS, MDIS, or whether a common unifying underlying chronic disorder may need to be excluded. For example patients with chronic inducible or chronic spontaneous urticaria (CSU) or asthma may develop urticaria/angioedema or wheeze during acute infections when oral non-steroidal anti-inflammatory drugs are administered together with different antibiotic classes and the infection acts as a co-factor (24). Up to 10% patients with CSU carry drug “allergy” labels, which often impede future medical treatments or therapies (25). Similarly, patients with mast cell activation syndromes may develop immediate hypersensitivity type reactions related to neuromuscular blocking agents rather than antibiotics administered during the perioperative period (26). Eczema flares triggered by acute infection and viral exanthems may also be difficult to differentiate from antibiotic related drug eruptions.

## Diagnostic workup

Diagnostic workup of suspected antibiotic allergies usually includes taking a thorough drug allergy history, followed by in-vivo or -vitro allergy tests, followed by a confirmatory challenge (if appropriate). Choosing the appropriate in-vivo or -vitro tests for each patient depends on the patient history and type of suspected DHR based on clinical suspicion. For in-vivo tests, immediate DHR are commonly confirmed with skin prick and intradermal tests while non-immediate reactions are confirmed with patch tests or intradermal tests with delayed reading. Although the performance of such in-vivo tests have been well established for penicillin, notable exceptions including poor negative predictive value of skin testing for piperacillin-tazobactam allergies, need to be emphasised (27). Furthermore, skin testing is not available for many antibiotics and not well studied except for penicillin. Negative in-vivo tests require a drug provocation test for confirmation. Drug provocation tests may be single or double blinded should the index reaction be associated with non-specific symptoms or signs (28). In-vitro tests measuring drug specific IgE are less commonly used in clinical practice because of the limited range of drugs commercially available, and suboptimal performance characteristics (sensitivity and specificity). For non-immediate reactions, lymphocyte transformation tests and ELISpot tests are usually only available in research centres or large tertiary referral centres (29). In patients with multiple antibiotic allergy where there are relative or absolute contraindications to drug provocation tests, combinations of in-vitro and in-vivo tests may be useful to identify the culprit antibiotic (30). However, the costs, availability and access to these tests need to be balanced with the need to delabel the multiple antibiotic allergies, the availability and risk-benefits of alternative potentially broader spectrum antibiotics. For instance, in tuberculous (TB) drug allergy, there are benefits of delabelling some of the first-line TB drugs implicated rather than proceed with second line TB treatment which may be less widely available, associated with more treatment related side-effects or result in prolonging the course of TB treatment (31, 32). Whether and how to proceed with further testing requires shared-decision making between the patient and the allergist.

## Efficacy of successful antibiotic allergy delabelling

Although there is not much data on delabelling multiple antibiotic allergies, the efficacy of delabelling individual allergies has been well demonstrated for a variety of different antibiotics. Prospective studies of patients following penicillin allergy delabelling has led to increase penicillin usage, reduction in non-penicillin antibiotic (such as fluoroquinolone) use, improved clinical outcomes, as well as reduce future healthcare costs (9, 13, 33–36). The positive impact of delabelling incorrect penicillin allergy labels have shown to be especially pronounced among susceptible populations such as the immunocompromised and elderly (37–40). Similar benefits have been observed upon delabelling of other non-penicillin antibiotics such as sulfa-antibiotics (41, 42). Although prospective data remains limited, the compound benefits following the delabelling of multiple antibiotic allergies will undoubtedly be exponential.

## Healthcare resource prioritisation in delabelling

In penicillin allergy delabelling, the use of nurse- and pharmacist-led protocol-driven services to delabel low-risk patients have been well-described (13, 14). Another model comprising evaluation of low risk penicillin allergy cases by non-allergists at spoke clinics within hospital departments of medicine, with training and support of an allergist-led hub has also been described (16). Such models have also been successful for other multi-disciplinary allergy initiatives (43, 44). These models of care may potentially be adapted for use in allergy/immunology services for patients with multiple antibiotic allergy where the number of provocation tests and patients who need to be tested far exceed the number of trained specialists in allergy/immunology. In any healthcare system with resource constraints, the patients requiring initiation of antibiotics earlier should be triaged and prioritised to be tested earlier e.g., bronchiectasis and primary immunodeficiency patients, rheumatology/haematology patients with recurrent infections.

## Cost-effectiveness of multiple antibiotic testing

Cost-effectiveness analyses evaluate whether a new health technology (test, device or therapeutic modality) provides value relative to other existing health technologies – in essence a comparison of costs and consequences (health outcomes) (45). In countries which do not have universal health care or where the healthcare system is co-payment or insurance based, patients may not be willing to pay for out-of-pocket expenses for multiple tests and evaluations for which they do not see any apparent benefit. Multiple visits for allergy tests are also associated with indirect costs e.g., time away from work, travelling time to the allergy clinic, time spent under observation, potential financial losses from absence from work. The cost of testing to the individual needs to be balanced against the cost to the healthcare system with the increased use of broad-spectrum antibiotics, risk of antimicrobial resistance and prolonged length of hospitalization should an antibiotic be needed. There have been few studies on the cost-effectiveness of delabelling as an allergy intervention, although these have been studied for multiplex allergen testing, adrenaline autoinjectors in anaphylaxis and venom immunotherapy (19, 46–48).

## Conclusion

True multiple drug allergy is far less common than MDIS or multiple incorrect allergy labels. Incorrect allergy labels may impact patient care and necessitate delabelling, it is imperative that inappropriate use of antibiotics in the community and in hospitals be curtailed through on-going physician- and patient-education. Any suspected drug allergy reaction when it occurs, should be promptly and accurately documented, a “drug allergy passport” or alert card be given to the patient, and electronic medical records linked to electronic decision support-alerting and prescribing systems updated (49, 50). These primary prevention interventions are probably the most important to prevent a future tsunami of more patients with multiple antibiotic allergy labels.
